# In vitro and in vivo anticancer studies of 2'-hydroxy chalcone derivatives exhibit apoptosis in colon cancer cells by HDAC inhibition and cell cycle arrest

**DOI:** 10.17179/excli2016-643

**Published:** 2017-04-03

**Authors:** Aditya Narayan Pande, Subhankar Biswas, Neetinkumar D. Reddy, B.S. Jayashree, Nitesh Kumar, C. Mallikarjuna Rao

**Affiliations:** 1 Department of Pharmacology, Manipal College of Pharmaceutical Sciences, Manipal University, Manipal-576104, Karnataka, India; 2 Department of Pharmaceutical Chemistry, Manipal College of Pharmaceutical Sciences, Manipal University, Manipal-576104, Karnataka, India

**Keywords:** chalcones, apoptosis, cell cycle, HDAC, DMH, colon cancer

## Abstract

Considering the therapeutic values of bioflavonoids in colon cancer treatment, six 2ʹ-hydroxy chalcones (C1-C6) were synthesized, characterized and screened for *in vitro* cytotoxicity on human colon carcinoma (HCT116) and African green monkey kidney epithelial cells (Vero). Only C5 showed selective cytotoxicity against HCT116 cells. Other potent cytotoxic compounds were C1, C2 and C3. Further screening included enzyme inhibition studies on histone deacetylase (HDAC) enzyme where C1 showed lowest IC_50_ value (105.03 µM). Based on cytotoxicity data C1, C2 and C3 were selected for further *in vitro* mechanistic studies, namely apoptotic studies (Acridine orange/Ethidium bromide (AO/EB) and Annexin V), cell cycle analysis using propidium iodide (PI) stain and *in vivo* anticancer efficacy in 1,2-dimethyl hydrazine (DMH) induced colorectal carcinoma in Wistar rats. The compounds induced apoptosis in more than 30 % cells in AO/EB and Annexin V staining. They also showed cell cycle arrest in G_2_/M phase with PI staining. They showed a significant reduction in aberrant crypt foci formation and adenocarcinoma count along with a significant (p<0.05) reduction in TNF-α levels as compared to DMH control at 100 mg/kg dose. Thus, it can be concluded that the synthesized 2ʹ-hydroxychalcones were effective against colon adenocarcinoma in *in vitro* and *in vivo* studies.

## Introduction

Cancer is a multifactorial disease characterized by uncontrolled and abnormal cellular growth. Unlike normal cells, cancer cells continue to grow and divide eventually replicating exponentially into harmful cells (Pisani et al., 1999[16]). It is undoubtedly a life threatening disease. However, there is a misconception about its cure. Much of the efforts in cancer research carried out during the last few decades prompted the researchers and cell biologist in understanding its pathophysiology, signaling mechanism and developing strategic therapies that can effectively treat cancer so as to eliminate or slow the impact of disease on patients' lives. The 5-year relative survival rate for all cancers diagnosed in 2004-2010 was 68 %, up from 49 % in 1975-1977 (Siegel et al., 2015[22]).

Among the various types of cancer diagnosed, it is worth mentioning that colorectal cancer (CRC) is the third most fatal malignancy affecting both men and women (Siegel et al., 2014[21]). Most CRC develops slowly as non-cancerous polyp (adenomas) and show harmful effects over years evolving into invasive cancer (Schulmann et al., 2002[20]). Several risk factors including age, familial history of adenomatous polyps, diet and heavy alcohol consumption have been implicated in the pathogenesis of CRC (Haggar and Boushey, 2009[6]). Apart from these factors, recent research has also highlighted the role of epigenetics in CRC (Goel and Boland, 2012[5]). Among the epigenetic factors, histone deacetylases (HDACs) a group of enzymes involved in silencing gene expression are reported to be over-expressed in CRC (Mariadason, 2008[11]). Amid the various modalities of treatment strategies available for CRC, surgery is the most preferred choice depending on the stage of CRC. However, a person's general health plays a crucial role for better outcome of surgery. Furthermore, surgery along with chemotherapy is recommended in many cases for long term survival and prevention of reoccurrence (Giacchetti et al., 1999[4]). Although there are many chemotherapeutic drugs for CRC, side effects associated with them limits their usage, providing researchers to develop newer molecules leading to better therapeutic outcome.

The current drug discovery program has explored natural products such as bio flavonoids, polyphenols, chalcones for their anti-oxidant and cytotoxic properties that have given insight to the medicinal chemists to use them as potential anti-cancer agents. Chalcones are precursors of flavonoids that are present in various parts of a plant having anti-inflammatory and anti-tumor activity (Jeon et al., 2012[7]; Zhang et al., 2013[26]). It is noteworthy to mention that quercetin, a cyclized chalcone and curcumin have been explored as potential anti-cancer agents. However, a few detailed studies have been carried out on the anti-tumor properties of chalcones against colon adenocarcinoma. They are considered to have fewer side effects when compared with chemotherapeutic agents (Syam et al., 2012[23]). In addition, there is an increasing arousal of interest in flavonols and other dietary poly phenols owing to their HDAC inhibitory activity in cancer cells (Rajendran et al., 2011[17]). Thus, chalcones and flavonols could be most appropriate candidates to be evaluated for their HDAC inhibitory potential. Hence, our study was aimed at developing different substituted chalcones and assessing their anticancer activity against colon adenocarcinoma. 

## Materials and Methods

### Chemicals and instruments

Chemicals used for synthesis were procured from Sigma-Aldrich Co. LLC, St. Louis, MO, USA; Merck Specialities Pvt. Ltd, Mumbai, India; Spectrochem Pvt. Ltd., Mumbai, MH, India. Melting point of the synthesized compounds were determined using capillary melting point apparatus from Toshniwal Systems and Instruments Pvt. Ltd., Chennai, TN, India. Thin layer chromatography was carried out on pre-coated silica gel plates procured from Merck # 60F254 using the following developing system: Hexane/ Ethyl acetate (8:2, v/v) and the spots were visualized under UV lamp (254 or 366 nm) and/or iodine vapor. The IR spectra were recorded using IR spectrometer (Model FTIR-8300, Shimadzu Co., Kyoto, Japan) using KBr pellets. Mass spectra were recorded using LC-MS (ESI) (Model LCMS-2010A, Shimadzu Co., Kyoto, Japan). ^1^H and ^13^C NMR were recorded at 400 MHz (Model Ascend 400, Bruker Biosciences Corporation, Billerica, MA, USA) using DMSO (D6) as solvent. Chemical shifts are reported in δ values (ppm). Signal multiplicities are represented by s (singlet), d (doublet), m (multiplet). All tested compounds possess a purity of not less than 95 %. 

### Synthesis

**Figure 1:**
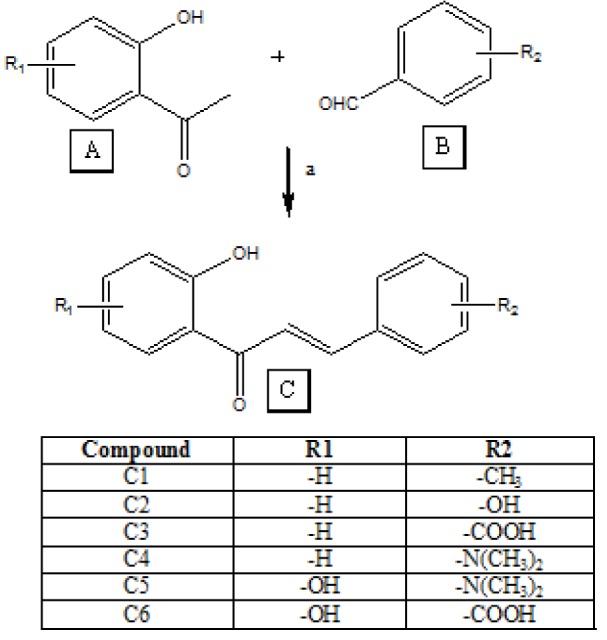
Synthetic scheme of chalcones. Synthesis of chalcones was carried out using acetophenone (A) and benzaldehyde (B) where 2ʹ-hydroxy acetophenone was used for the preparation of C1-C4, 2,4-dihydroxy acetophenone for C5 and 2,6-dihydroxy acetophenone for C6. Benzaldehyde used were 4-methyl benzaldehyde for C1, 4-hydroxy benzaldehyde for C2, 4-carboxy benzaldehyde for C3 and C6 and 4-(dimethylamino)benzaldehyde for C4 and C5. The reaction conditions were (a) 20 % w/v potassium hydroxide, ethanol and stirring at room temperature for 12-16 h.

Compounds were synthesized by Claisen-Schmidt condensation reaction. To a solution of substituted 2-hydroxyacetophenone (5 mM) in 15 ml of ethanol, 5 ml of 20 % aqueous potassium hydroxide was added. After stirring the reaction mixture for 30 minutes, substituted benzaldehyde (5 mM) was added in portions. Stirring was continued for a period of 10 h or more till the completion of reaction. The progress of the reaction was monitored by TLC using n-hexane and ethylacetate (8:2). The mixture was then suspended in ice cold water and the resulting solution was acidified with dilute hydrochloric acid. The precipitated product was collected by filtration and washed with ice cold water to remove any colored impurities and dried. It was crystallized using ethanol (Ameta et al., 2011[1]). The synthesis scheme is shown in Figure 1(Fig. 1).

#### Physiochemical and spectral data of the synthesized substituted chalcones

##### C1: 1-(2-Hydroxy-phenyl)-3-p-tolyl-propenone 

Yield = 80 %, m.p. 168 ± 2 °C uncorrected; IR (KBr); 3444.98 (-OH, Str), 1639 (C=O, Str), 2918 (Ar, C-H) cm^-1^; 1H NMR (400 MHz, DMSO (D6)) δ 12.56 (1H, s), 8.25 (1H, d), 8.013 (1H, d) 7.845-7.55 (4H, m), 7.31-6.99 (4H, m), 2.36 (3H, d) ppm; ^13^C NMR (400 MHz, DMSO (D6)) δ 194.14 (C=O), 162.08 (C-OH), 145.44 (C=C-C), 143.0 (C-CH_3_), 141.67-118.20 (10C-C), 21.60 (C-C-H) ppm. MS (ESI): m/z (M+1) 239.17 

##### C2: 3-(4-Hydroxy-phenyl)-1-(2-hydroxy-phenyl)-propenone 

Yield = 75 %, m.p. 172 ± 2 °C uncorrected; IR (KBr); 3441.12(-OH, Str), 1633.76 (C=O, Str), 1082 (C-O, Str) cm^-1^; ^1^H NMR (400 MHz, DMSO (D6)) δ 14.63 (1H, s), 9.626 (1H, s), 7.798 (1H, d), 7.591 (1H, d), 7.358-6.816 (6H, m), 5.566 (2H, d) ppm; ^13^C NMR (400 MHz, DMSO (D6)) δ 192.45 (C=O), 158.17 (OH-C=C), 136.68 (C=C-OH), 129.56 (C-C=C), 128.80-115.63 (11 C-C) ppm. MS (ESI): m/z (M+1) 241.0

##### C3: 4-[3-(2-Hydroxy-phenyl)-3-oxo-propenyl]-benzoic acid 

Yield = 96 %, m.p. 180 ± 2 °C uncorrected; IR (KBr); 3433 (-OH, Str), 1633 (C=O, Str), 1766 (Ar COOH, C=O Str), 2924.18 (COOH, O-H Str) cm^-1^; ^1^H NMR (400 MHz, DMSO (D6)) δ 12.35 (1H, s), 11.53 (1H, s), 7.822 (1H, d), 7.862 (1H, d), 7.821-7.513 (4H, m), 7.008 -6.117 (4H, m) ppm; ^13^C NMR (400 MHz, DMSO (D6)) δ 201.45 (C=O), 170.18 (OH-C=O), 162.10 (C-C=C), 136.66-118.14 (12C-C) ppm. MS (ESI): m/z (M+1) 269.08

##### C4: 3-(4-Dimethylamino-phenyl)-1-(2-hydroxy-phenyl)-propenone

Yield = 75 %, m.p. 178 ± 2 °C uncorrected; IR (KBr); 3448 (-OH, Str), 1604 (C=O, Str), 1377 .22 (CH3)

##### C5: 1-(2-Hydroxy-4-methyl-phenyl)-3-p-tolyl-propenone

Yield = 80 %, m.p. 153 ± 2 °C uncorrected; IR (KBr); 3443.05 (-OH, Str), 1597 (C=O, Str)

##### C6: 4-[3-(2,6-Dihydroxy-phenyl)-3-oxo-propenyl]-benzoic acid 

Yield = 65 %, m.p. 186 ± 2 °C uncorrected; IR (KBr); 3070.78 (-OH, Str), 1616.40 (C=O, Str), 1707.06 (Ar COOH, C=O Str), 2652.21 (COOH, O-H Str).

### In vitro studies 

#### Cell lines and their maintenance 

Human colorectal carcinoma (HCT116) and African green monkey kidney epithelial cells (VERO) were procured from the National Centre for Cell Science, Pune, MH, India. The cells were maintained in Dulbecco's Modified Eagle's Medium (DMEM) (Sigma-Aldrich Co. LLC, ST. Louis, Mo, USA) supplemented with 10 % Fetal Bovine Serum (FBS) (HiMedia Laboratories, Mumbai, India) and 1 × Penicillin/Streptomycin at 37 °C in CO_2_ incubator (NU-5501 E/G, NuAire Inc., Plymoth, MN, USA) in humidified atmosphere of 5 % CO_2_ and 95 % air. The cells were maintained by routine sub-culturing in 25 cm^2^ tissue culture flasks. 

#### Cytotoxicity assay 

Cytotoxic potential of the test compounds were assessed using MTT assay (Kumar et al., 2016[9]). In brief, HCT116 and Vero cells were harvested from confluent flask and seeded (5 × 10^4 ^cells/well) in 96 well plates. After 24 h of incubation, the cells were exposed to different concentration of the test compounds for 48 h. Further, 50 µl of MTT reagent (HiMedia Laboratories, Mumbai, India) (2 mg/ml in sterile PBS) was added into each well after 48 h and incubated for 3 more h. The formazan crystals formed were solubilized using 100 % DMSO and the optical density was measured at 540 nm using micro plate reader (ELx800, BioTek Instruments Inc., Winooski, VT, USA). 

#### Whole cell HDAC enzyme assay

HCT116 cells were harvested and seeded in 96 well sterile, black well plates at 2 × 10^4^ cells/well and incubated overnight. Further, the cells were treated with different concentration of the test compounds for a period of 18 h. Then, 15 mM Boc-Lys(Ac)-AMC substrate (Sigma Aldrich # SCP0168) was added and incubated for 1 h. The reaction was terminated by the addition of 50 µL of stop solution (trypsin 2 mg/ml, 1 % NP40, 1 µl SAHA) in HDAC assay buffer (25 mM Tris-HCl (pH 8.0), 137 mM NaCl, 2.7 mM KCl, 1 mM MgCl_2_). The reaction was then allowed to proceed for 15 min at 37 °C after which, the fluorescence was measured at 360 nm excitation and 460 nm emission using fluorescence micro plate reader (FLx800, BioTek Instruments Inc., Winooski, VT, USA) (Reddy et al., 2015[18]).

#### Acridine orange/Ethidium bromide (AO/EB) staining

AO/EB dual staining was performed in order to determine the apoptosis inducing potential of the selected test compounds in HCT116 cell line with few modifications (Kumar et al., 2016[9]). In brief, 5 × 10^5^ cells were seeded in 6 wells plate containing 2 ml of medium and incubated for 24 h. After 24 h, cells were treated with the test compounds and incubated for 48 h. After 48 h of incubation, the wells were washed with phosphate buffer saline (PBS) and cells were fixed with ice cold ethanol (70 %) for 30 min. Ethanol was then removed and cells were washed again with PBS followed by the addition of 200 µl of AO/EB (20/30 µg/ml) stain in each well. The plate was later kept in the dark for 20 min. The excess stain was further washed thrice with PBS and observed for their fluorescence under a fluorescent microscope (Eclipse TS100-F, Nikon Instruments Inc., Melville, NY, USA).

#### Annexin V assay

Apoptosis determination by Annexin V staining was carried out using Muse Cell Analyzer with the kit provided by the manufacturer Merck Millipore. In brief, 1 × 10^6^ cells were seeded in 60 mm tissue culture dish and after overnight adherence test compounds were added and incubated. After 48 h the cells were detached by trypsinization, centrifuged and resuspended. 100 µl of cell suspension was added with 100 µl of Annexin V reagent and incubated for 20 min at room temperature following which the cells were analyzed for apoptosis.

#### Cell cycle analysis

The ability of the test compounds to arrest any phase of the cell cycle was determined using the described procedure (Reddy et al., 2015[18]). In this method, HCT116 cells were harvested and seeded at a density of 1 × 10^6^ cell in 60 mm petri plate and incubated for 24 h. After 24 h cells were treated with test compounds for 48 h. Then the cells were washed with PBS, trypsinized and centrifuged. The cell pellets were later fixed in 70 % ice cold ethanol and stored at -20 °C for 24 h. After fixing, the pellet was dislodged in PBS and stained with propidium iodide solution. The cells were then analyzed using Accuri C6 flow cytometer with the threshold levels adjusted to remove the debris (BD Biosciences, San Jose, CA, USA) and data analysis were performed using BD Accuri™ C6 software. 

### In vivo studies

#### Animals

Male Wistar rats inbred at the Central Animal Research Facility, Manipal University, were used in our study. The animal care and handling were carried out in accordance with the guidelines issued by the Institutional Animal Ethics Committee (IAEC), Manipal. After obtaining research proposal approval (IAEC/KMC/16/2015) the animals were acclimatized to the experimental room having temperature of 23 ± 2 °C, humidity (50 ± 5 %) and 12 h light and dark cycles. Rats were housed in sterile polypropylene cages containing sterile paddy. 

#### Acute toxicity studies 

Acute toxicity study was carried out to determine the safe dose using Organization for Economic Cooperation and Development (OECD) - 425 guideline. Limit test was performed using 2000 mg/kg dose of the test compounds in 6 h fasted rats. Animals were observed for any signs of toxicity for the first 4 h continuously and then daily for 14 days. 

#### Preparation of the test compound and standard drug

Test compound: Three test compounds, namely C1, C2 & C3 were suspended in 0.25 % sodium carboxy methyl cellulose (CMC) and were administered orally *(p.o)* with a dosing volume of 10 ml/kg. 

Standard drug: 5-Fluorouracil (5-FU) was used as standard. It was procured in the form of injection and administered intraperitoneally *(i.p)* with a dosing volume of 10 ml/kg.

#### DMH (1,2-dimethyl hydrazine) induced colon cancer in Wistar rats

Induction of colon cancer was achieved using DMH according to a previously described procedure with few modifications (Perše and Cerar, 2005[15]). DMH at a dose of 30 mg/kg was administered *i.p* once a week for 20 weeks. The incidence of aberrant crypt foci (ACFs) and adenocarcinoma were confirmed by sacrificing a few of the animals after 20 weeks confirming the induction of colon cancer in experimental animals. Finally, the animals were randomized into five experimental groups based on their body weight. 

#### Experimental groups

Group 1 (normal control): Animals (n=6) were administered 0.25 % CMC in water p.o.

Group 2 (DMH control): Animals (n=6) were administered DMH

Group 3 (standard drug): Animals (n=6) received 5-FU 10 mg/kg i.p. for 21 days of study period

Group 4 (C1): Animals (n=6) received C1 at 100 mg/kg p.o for 21 days

Group 5 (C2): Animals (n=6) received C2 at 100 mg/kg p.o. for 21 days. 

Group 6 (C3): Animals (n=6) received C3 at 100 mg/kg p.o. for 21 days. 

### Parameters assessed in the experimental animals

#### ACF formation and adenocarcinoma incidence

The distal part of the colon, which was removed from the experimental animals after the study period was cut open and placed flat on a filter paper and fixed with 10 % buffered formalin for 12 h. Further, it was stained with 0.1 % methylene blue in PBS for 5 min. Specimens were later observed under microscope for ACF formation and were calculated as the number of counts/5 cm^2^ in colon tissue. The entire colon was considered to study the incidence of adenocarcinoma. The growth was critically observed and the count and size were noted from each animal. 

#### TNF-α level

The levels of TNF-α were estimated in the colon of experimental animals, for which 10 % homogenate of colon tissue was prepared in tissue lysis buffer. The homogenate was centrifuged and the supernatant was collected to measure TNF-α levels using commercially available ELISA kits of rat TNF-α (# KRC3011, Invitrogen).

#### Colon length/weight ratio and organ index

Length of the isolated colon was measured in centimeters and weight was measured in g. The colon length/weight (L/W) ratio was then calculated. Isolated spleen, kidney and heart of the experimental animals were also weighed in g and respective index was calculated.

#### Histopathology of colon

Histopathology was carried out according to the described procedure (Reddy et al., 2015[18]). The stained slides were then analyzed under a microscope for any anatomical changes. 

#### Statistical analysis

All the values were expressed as mean ± SEM of 6 animals. Data were analyzed using one-way ANOVA followed by Tukey's multiple comparison tests using Prism 5.03 (Graph Pad Software Inc., La Jolla, CA, USA). Values of *p* < 0.05 were considered to be significant. 

## Results

### In vitro studies

#### MTT assay

MTT assay was carried out to evaluate the cytotoxic potential of the synthesized compounds on HCT116 and Vero cell line. Compounds C1, C2, C3 and C5 were found to be cytotoxic against colon cancer cell line after 48 h of treatment. Among these compounds, C1 was found to be most cytotoxic with an IC_50_ value of 37.07 µM on HCT116 cells. Furthermore, in Vero cell lines C1, C2 and C3 exhibited potential cytotoxicity compared with the remaining three synthesized compounds. Moreover C1 displayed greater cytotoxicity compared with C2 and C3. Table 1(Tab. 1) provides the IC_50_ value of the synthesized compounds on both the cell lines tested. 

#### Whole cell HDAC assay

**Figure 2:**
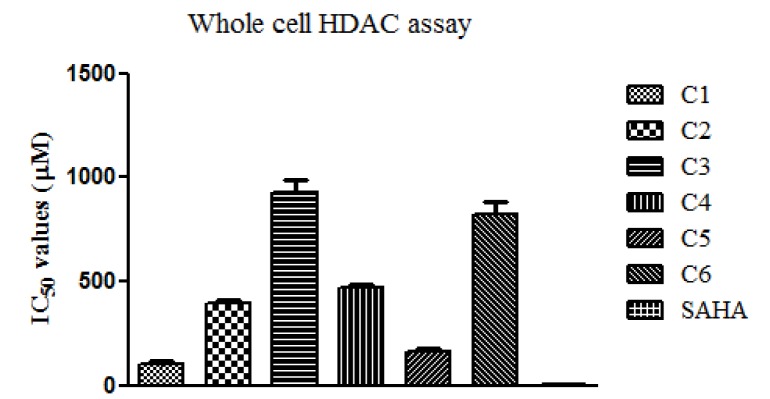
Whole cell HDAC inhibition assay. The histogram plot represents the IC_50_ value of whole cell HDAC enzyme inhibition assay carried out in HCT116 cell line. All values are represented as mean ± SEM and the experiments were carried out in triplicate.

The whole cell HDAC enzyme inhibition assay was performed to determine the effect of test compounds on the epigenetic machinery of HCT116 cells. Dose dependent HDAC enzyme inhibition was observed in all treatment groups, with C1 as the most potent compound with an IC_50 _value of 105 ± 10 µM. The other potent compound was found to be C5 with IC_50_ value below 200 µM *i.e,* 160.4 ± 15.5 µM. The IC_50 _values for the remaining compounds were 394.3 ± 12.9, 928.7 ± 56.6, 470.4 ± 11 and 826.1 ± 58.1 µM respectively for C2, C3, C4 and C6. SAHA, a non-specific HDAC inhibitor, was used as standard and was found to have an IC_50_ value of 3.6 ± 0.2 µM. Figure 2(Fig. 2) shows the histogram plot of whole cell HDAC assay.

#### AO/EB staining

**Figure 3:**
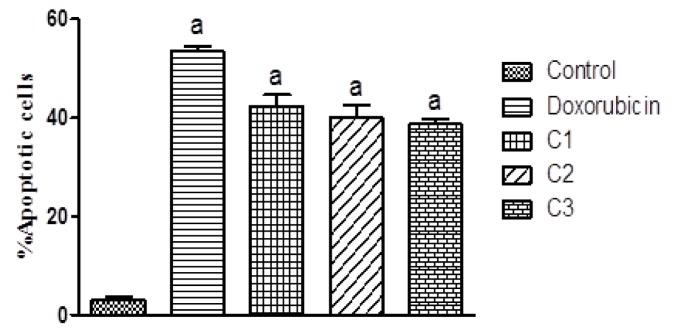
Percentage apoptotic cells in HCT116 cell line. Percentage apoptotic cells in HCT116 cell line determined by AO/EB staining. All values are mean ± SEM of 100 cells. ^a^p< 0.05 vs. control.

**Figure 4:**
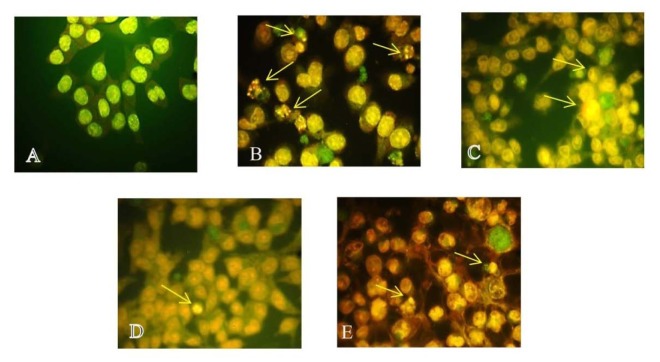
AO/EB staining in HCT116 cell line. The images indicate induction of apoptosis in HCT116 cell line by various treatment groups after 48 hrs. (A) Normal control, (B) 5-FU, (C) C1, (D) C2, (E) C3. Arrows indicate condensed nucleus. Magnification 40X.

To determine the mechanism of cell death induced by test compounds, fluorescent dye based apoptosis assay was carried out. In the present study Acridine Orange/Ethidium Bromide stain was used. Green stained unfragmented nuclei were observed in untreated cells, indicating non-apoptotic cells. Whereas, the presence of highly condensed chromosome was visible in cells treated with test compounds appearing as green fragmented nuclei. Treatment with 5-FU showed 45 ± 0.87 % apoptotic cells in HCT116 cells. Alternatively, % apoptotic cells in C1, C2 and C3 treated groups were found to be 42.4 ± 2.25 %, 40 ± 2.5 % and 38.7 ± 0.94 % respectively. Figure 3(Fig. 3) shows the % apoptotic cells represented as histogram and Figure 4(Fig. 4) shows the fluorescent images. 

#### Apoptosis detection study

**Figure 5:**
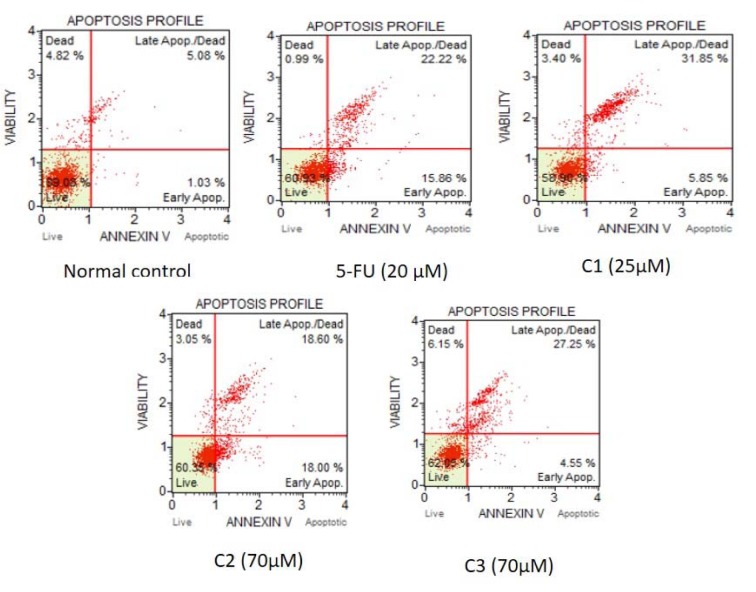
Determination of apoptosis by Annexin V staining. Effect of binding of Annexin V on the surface of HCT116 cell after 48 hrs of treatment. Apoptosis was determined as % live, early and late apoptosis/dead cells by MUSE cell analyzer.

During apoptosis, phosphatidylserine which is predominantly situated along the cytosolic side of the plasma membrane translocates to the extracellular side. After translocation, it is detectable by the family of calcium-dependent phospholipid binding proteins called Annexin. More than 30 % of apoptotic cells were observed after 48h of incubation in the various treatment groups. Total apoptotic cells in normal, 5-FU, C1, C2 and C3 was found to be 6.10 %, 38.08 %, 37.70 %, 36.60 %, and 31.80 % of total cells respectively. Early apoptotic events were more prominent in the treatment of 5-FU and C2 with 15.86 % and 18 % of total cells, respectively, while it was less in C1 and C4 treatment 5.85 % and 4.55 %, respectively (Figure 5(Fig. 5)).

#### Cell cycle analysis

**Figure 6:**
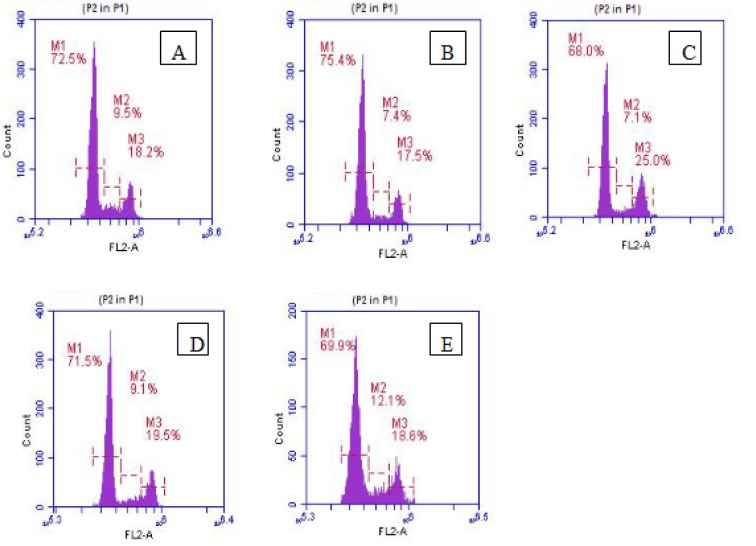
Cell cycle analysis in HCT116 cell line. Effect on the cell cycle of HCT116 cells after 48 hrs of treatment. Histogram showing various phases of the cell cycle where M1 represent G_0_/G_1_ phase, M2 represent S phase and M3 represent G_2_/M phase. (A) Normal control, (B) Doxorubicin, (C) C1, (D) C2, (E) C3

Cell cycle analysis revealed that cells in normal control contained 72.5 %, 9.5 % and 18.2 % cells in G_0_/G_1_, S and G_2_/M phase respectively. The treatments with C1 and C2 showed a notable cell cycle arrest in G_2_/M phase by increasing the percentage cell counts compared to normal control *i.e.,* 25 % and 19.5 % cells respectively. These results suggested potentials of C1 and C2 as a G_2_/M phase blocker. Furthermore an increase in the percentage of cells in S phase (12 %) was observed in C3 treatment compared to normal control (Figure 6(Fig. 6)).

### In vivo studies

#### In vivo toxicity studies

No signs of toxicity were observed in the experimental animals treated with the test compounds at 2000 mg/kg dose. Further studies were carried out using 1/20^th^ of the administered dose. 

#### DMH (1,2-dimethyl hydrazine) induced colon cancer in Wistar rats 

##### ACF formation and adenocarcinoma incidence

The incidence of colon carcinoma is commonly detected with the presence of aberrant crypt foci and the incidence of adenocarcinoma. The ACF formation in the DMH treated control group were 81.6 ± 1.4/5 cm^2 ^of colon tissue, whereas no such incidences were observed in normal control group. The ACF count was found to be significantly (p<0.05) lower in the all treatment groups compared to DMH control (Table 2(Tab. 2)). 

Adenocarcinoma formation was observed in the entire colon region of DMH treated control group (15 ± 1.2). Treatment with 5-FU and C1 showed significant decrease in the number of adenocarcinoma compared to DMH control group (7 ± 0.5 and 10 ± 0.8 respectively). However no significant decrease in adenocarcinoma was observed in C2 and C3 treatment compared to DMH control group (Table 2(Tab. 2)). 

##### TNF-α level in colon homogenate

**Figure 7:**
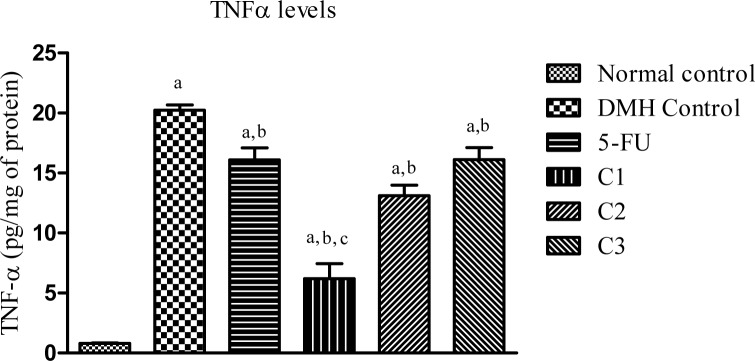
Effect of various treatments on TNF-α levels. Effect of DMH administration for 21 days on the inflammatory marker TNF-α in colon tissue homogenate. All values are mean ± SEM of 6 animals. ^a^p<0.05 vs. Normal control, ^b^p<0.05 vs. DMH control, ^c^p<0.05 vs. 5-FU.

A significant (p<0.05) increase in the levels of TNF-α was observed in DMH control group (20.2 ± 0.8) compared to normal control (0.8 ± 0.1) group, suggesting an increase in inflammation. 5-FU treatment (16.09 ± 1.9) was able to reverse the increased TNF-α levels in the colon homogenate when compared with the DMH control group. Treatment with all test compounds showed significant (p<0.05) reversal in the increased TNF-α levels compared to DMH control group. Among the tested compounds, the maximum reversal in TNF- α levels was observed in C1 treated group (6.18 ± 2.5), which was found to be significant compared to DMH control group and 5-FU group (Figure 7(Fig. 7)).

##### Colon length/weight ratio and organ index

Formation of adenomas or polyps in the colon causes a decrease in the length of colon with an increase in its weight. We observed a significant (p<0.05) increase in colon length/weight ratio in the DMH control group compared with that of normal control group. No significant (p<0.05) decrease in length/ weight ratio was observed by the treatment with 5-FU and C3 compared to DMH control group. However, treatment with C1 and C2 significantly lowered the Colon L/W ratio compared to DMH control group (Table 3(Tab. 3)). There were no significant alterations in various organ indices in the treatment groups compared to DMH and normal control (Table 4(Tab. 4)). 

##### Histopathology of colon

**Figure 8:**
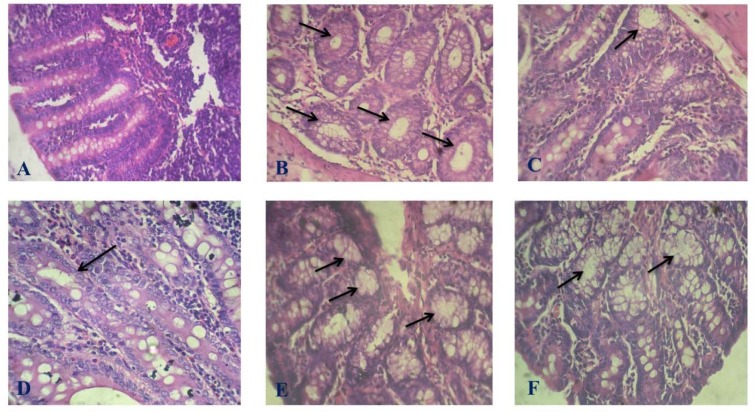
Effect of test compounds on the histopathology of colon. Photomicrographs of histological changes in the colon of experimental animals at 400 x magnification. (A) Normal Control, (B) DMH control, (C) 5-FU, (D) C1, (E) C2, (F) C3. The arrows indicate aberrant crypt foci.

Observation of the section of colon in the normal control group displayed a normal architecture with finger like mucosal projections called villi and the presence of crypts in between them. No sign of dysplasia or crypt abscess was observed in the normal control group. However, sections of colon in DMH control group had a distorted morphology with the formation of crypt abscess and aberrant crypt foci. Treatment with various compounds showed a restoration in the morphology of the colon along with a reduction in the formation of crypt abscess (Figure 8(Fig. 8)).

## Discussion

In the present study, six substituted 2ʹhydroxy chalcones were synthesized and their anticancer potential was evaluated using *in vitro* mechanistic and target specific studies in human colon cancer cell line. Furthermore, the efficacy of the test compounds was assessed in *in vivo* model of colon adenocarcinoma. Since, various factors (including genetics, epigenetics and environmental) are involved in understanding the pathophysiology of cancer, it is regarded as a multifactorial disease (Liu et al., 2008[10]). Several targets are validated among these factors which play a critical role in this complex disease. HDACs, a group of enzymes, are involved in altering tumor suppressor gene expression and regulating the stages of apoptosis and cell cycle progression (Minucci and Pelicci, 2006[12]). Numerous studies have demonstrated the increased expression of HDAC in colon cancer (Nakagawa et al., 2007[13]), based on which we explored the ability of the synthesized compounds to inhibit this target.

Cytotoxicity assays are frequently used to screen various compounds for their ability to inhibit cell proliferation and viability. One colon cancer cell line, namely HCT116 and one normal cell line, i.e., Vero cells were used to evaluate the cytotoxic potential of these synthesized compounds. On HCT 116, C1, C2, C3 showed IC_50_ values below 200 µM. Except C5, none of the tested compounds showed selective cytotoxicity to cancer cells. 

Further HDAC inhibition study was performed for all the synthesized compounds to evaluate their potential to modulate the epigenetic pathways leading to the expression of apoptosis inducing genes. It is well documented that cancer development is not limited to genetic changes rather it involves interplay between genetic and epigenetic regulation (You and Jones, 2012[25]). The insight on the epigenetic regulation provides a platform to understand the rationale for the development of drug candidates that could target the epigenome of a cell. Further, the chances of interaction between histone and DNA increases owing to the deacetylation of histone resulting in chromatin compaction and repression of genes involved in apoptosis and cell cycle progression (Ropero and Esteller, 2007[19]). Moreover, an imbalance between the enzyme histone acetyltransferases (HAT) and histone deacetylases (HDAC) is observed in cancer with the balance shifting towards HDAC over activity. Thus, the present study was designed to identify the dose dependent inhibitory response of the synthesized test compounds on HDAC enzymes. Four compounds namely C1, C2, C4 and C5 showed inhibition of HDAC with IC_50_ value below 500. Thus taking mainly cytotoxicity data into consideration along with HDAC inhibition data three molecules were selected for further evaluation of their mechanistic and efficacy study.

Attempts were made to understand the Structure Activity Relationship (SAR) of the chalcones synthesized. Although we could not clearly arrive at the SAR of the synthesized compounds, some deductions from the structural modifications that might have directly or indirectly contributed for eliciting the anti-cancer activity could be drawn. Out of the six test compounds synthesized and screened for cytotoxicity compounds C1, C2 and C3 with 2ʹ-hydroxy group and methyl, hydroxyl and carboxy substitution at the 4^th^ position of the B ring, respectively, were found to exhibit anti-cancer potential at an IC_50_ value of less than 200 µM when compared with the other synthesized compounds. The 2ʹ-hydroxyl group seems to have played a significant role in establishing the structural activity of chalcones with respect to mostly stabilizing them by forming hydrogen bond. Further, the 2ʹ-hydroxy group might also play a crucial role in chalcone-flavonone equilibrium (Avila et al., 2008[2]). For this reason, the 2ʹ-hydroxy group might be considered as an important functional group contributing towards the activity.

 Many reports available on chalcones showing anti-cancer potential bears a substitution at the para position on the phenyl ring. Thus, the position 4 of B-ring plays an important role in determining the anti-cancer activity. In our study, we observed that when para position is substituted with methyl, hydroxyl and carboxyl group, the chalcones have shown promising anticancer activity. However, substitution with other group such as para dimethylamino leads to less active compounds.

Further substitution bearing additional hydroxyl group at 4ʹ and 6ʹ position of A ring along with para dimethylamino and carboxyl substituent at the 4^th^ position of the B ring seems to have generated compounds with lower activity than when they were not substituted. The mechanism of cell death and the arrest of cell cycle are the important parameter for assessing anticancer potential of any drug. Thus, we examined the nature of the cell death induced by them using dual staining. AO/EB staining showed a significant change in nucleomorphological changes compared to the control cells. These changes were represented in the form of increase in apoptotic index. To further confirm these visual changes, the flow cytometry study was performed using Annexin V stain, which reflected two distinct ways through which cells probably die: apoptotic and necrotic pathways. Most of the potential anticancer drugs would kill cells by inducing apoptosis (Kaufmann and Vaux, 2003[8]). All three tested compound showed more than 30 % cells underwent apoptotic changes in cells which were significantly higher than the normal control, indicating that the nature of cell death was mediated by apoptotic pathway. 

Cell cycle analysis was based upon the DNA content of a cell through flow cytometer, where cells in various phases (G_0_/G_1_, S and G_2_/M) of the cell cycle were estimated. Further, we could assess whether the test compounds were cell cycle specific inhibitors or not. G_1_ is the initial phase of the cell cycle where, DNA damage would terminate the progression of the cell cycle and allows repair to take place before cell would enter the S phase. In the event of unsuccessful repair, accumulation of phosphorylated p53 initiates for the programmed cell death (Nowsheen and Yang, 2012[14]). Our results suggested that the test compounds were able to induce apoptosis and inhibit cell division mostly by arresting G_0_/G_1_ phase of the cell cycle. 

Further, development of colorectal cancer in rodents was observed using DMH, a highly specific colorectal carcinogen. DMH and its metabolite azoxymethane (AOM) promotes the initiation and advancement of colorectal carcinogenesis in rodents. Aberrant crypt foci are the first identifiable colonic lesions. A similar observation was found in our study confirming the induction of colorectal carcinoma in experimental animals. Literature suggests that, the reduction in ACF is a marker of recovery from colorectal carcinoma (Davis and Iwahashi, 2001[3]). Our results demonstrated that the test compounds were able to reduce ACF formation significantly when compared to DMH control suggesting their preventive role. Alternatively, a reduction in the number of colon adenocarcinoma in the treatment groups further supported their effectiveness. Colonic edema due to tissue injury or inflammation could also cause a rise in colon weight/length ratio. Our treatment reduced the rise in colon weight/length ratio when compared with that of DMH control further suggesting their role in lowering the tissue injury or inflammation. This was further supported by the estimation of TNF-α level in colonic homogenate. TNF-α is a well-established marker for inflammatory pathway and their levels are increased in DMH exposed rats (Umesalma and Sudhandiran, 2010[24]). Here, the test compounds were able to decrease TNF-α level in DMH treated rats, indicating their ability to counteract inflammation arising from tissue injury. Histopathological studies showed aberrant crypt foci formation and mucosal enlargement in DMH treated group. Restoration in the morphology of colon was also evident in the treatment groups with reduction in mucosal enlargement and reduced ACF formation suggesting their protective effect. 

The present study was designed to evaluate the protective effect of various substituted chalcones both *in vitro* and *in vivo*. *In vitro* studies proved the potential of the compounds to induce apoptosis and arrest G_0_/G_1_ phase of cell cycle in human colon cancer cell line. We also found that they were able to alter the epigenetic pathways evident from HDAC inhibition. In addition, we observed a reduction in ACF and adenocarcinoma formation in the colon of animals treated with the test compounds. Furthermore, they were able to lower the increased levels of TNF-α suggesting their role in inflammation. These results demonstrated the efficacy of the compounds against colon adenocarcinoma, providing a potential lead for anticancer drug development.

## Notes

Aditya Narayan Pande and Subhankar Biswas contributed equally to this work.

## Acknowledgements

The work was supported by a Grant SR/SO/HS-0282/2012 obtained from the Science and Engineering Research Board, Department of Science and Technology (DST-SERB). The authors would like to acknowledge All India Council for Technical Education (AICTE) for providing funds for the procurement of Flow cytometer used in the study. We thank the Department of Pharmacology, Manipal College of Pharmaceutical Sciences, Manipal University, India for providing necessary facilities to carry out the present work.

## Conflict of interest

The authors declare that they have no conflict of interest to disclose.

## Figures and Tables

**Table 1:**
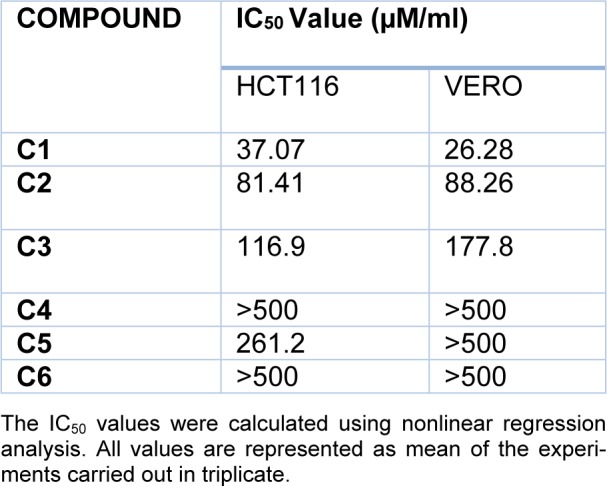
*In vitro *cytotoxicity screening in human colon cancer and normal cells

**Table 2:**
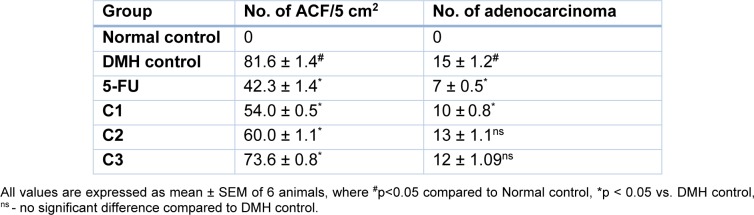
Effect of DMH and test compounds on ACF and adenocarcinoma count

**Table 3:**
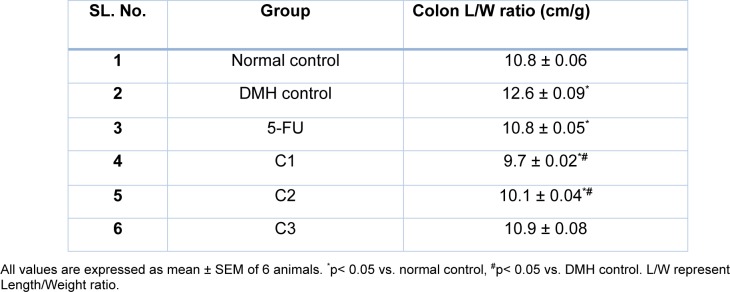
Effect of various treatments on colon L/W ratio

**Table 4:**
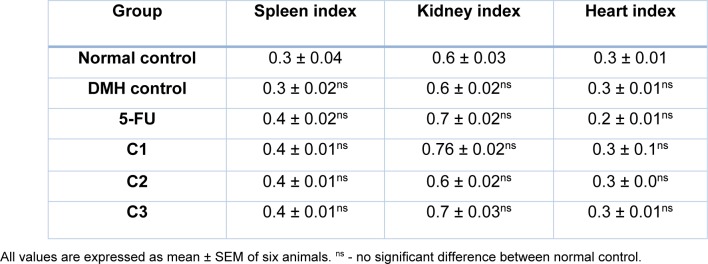
Effect of test compounds on organ index

## References

[1] Ameta K, Rathore NS, Kumar B (2011). Synthesis of some novel chalcones and their facile one-pot conversion to 2-aminobenzene-1, 3-dicarbonitriles using malononitrile. Analele Universitatii Bucuresti Chimie.

[2] Avila HP, Smânia Ede F, Monache FD, Smânia A (2008). Structure-activity relationship of antibacterial chalcones. Bioorg Med Chem.

[3] Davis PA, Iwahashi CK (2001). Whole almonds and almond fractions reduce aberrant crypt foci in a rat model of colon carcinogenesis. Cancer Lett.

[4] Giacchetti S, Itzhaki M, Gruia G, Adam R, Zidani R, Kunstlinger F (1999). Long-term survival of patients with unresectable colorectal cancer liver metastases following infusional chemotherapy with 5-fluorouracil, leucovorin, oxaliplatin and surgery. Ann Oncol.

[5] Goel A, Boland CR (2012). Epigenetics of colorectal cancer. Gastroenterology.

[6] Haggar FA, Boushey RP (2009). Colorectal cancer epidemiology: incidence, mortality, survival, and risk factors. Clin Colon Rectal Surg.

[7] Jeon J-H, Kim S-J, Kim C-G, Kim J-K, Jun J-G (2012). Synthesis of biologically active chalcones and their anti-inflammatory effects. Bull Korean Chem Soc.

[8] Kaufmann SH, Vaux DL (2003). Alterations in the apoptotic machinery and their potential role in anticancer drug resistance. Oncogene.

[9] Kumar H, Savaliya M, Biswas S, Nayak PG, Maliyakkal N, Manjunath Setty M (2016). Assessment of the in vitro cytotoxicity and in vivo anti-tumor activity of the alcoholic stem bark extract/fractions of Mimusops elengi Linn. Cytotechnology.

[10] Liu L, Li Y, Tollefsbol TO (2008). Gene-environment interactions and epigenetic basis of human diseases. Curr Issues Mol Biol.

[11] Mariadason JM (2008). HDACs and HDAC inhibitors in colon cancer. Epigenetics.

[12] Minucci S, Pelicci PG (2006). Histone deacetylase inhibitors and the promise of epigenetic (and more) treatments for cancer. Nat Rev Cancer.

[13] Nakagawa M, Oda Y, Eguchi T, Aishima S, Yao T, Hosoi F (2007). Expression profile of class I histone deacetylases in human cancer tissues. Oncol Rep.

[14] Nowsheen S, Yang E (2012). The intersection between DNA damage response and cell death pathways. Exp Oncol.

[15] Perše M, Cerar A (2005). The dimethylhydrazine induced colorectal tumours in rat-experimental colorectal carcinogenesis. Radiol Oncol.

[16] Pisani P, Parkin DM, Bray F, Ferlay J (1999). Estimates of the worldwide mortality from 25 cancers in 1990. Int J Cancer.

[17] Rajendran P, Ho E, Williams DE, Dashwood RH (2011). Dietary phytochemicals, HDAC inhibition, and DNA damage/repair defects in cancer cells. Clin Epigenetics.

[18] Reddy ND, Shoja MH, Jayashree BS, Nayak PG, Kumar N, Prasad VG (2015). In vitro and in vivo evaluation of novel cinnamyl sulfonamide hydroxamate derivative against colon adenocarcinoma. Chem Biol Interact.

[19] Ropero S, Esteller M (2007). The role of histone deacetylases (HDACs) in human cancer. Mol Oncol.

[20] Schulmann K, Reiser M, Schmiegel W (2002). Colonic cancer and polyps. Best Pract Res Clin Gastroenterol.

[21] Siegel R, DeSantis C, Jemal A (2014). Colorectal cancer statistics, 2014. CA Cancer J Clin.

[22] Siegel RL, Miller KD, Jemal A (2015). Cancer statistics, 2015. CA Cancer J Clin.

[23] Syam S, Abdelwahab SI, Al-Mamary MA, Mohan S (2012). Synthesis of chalcones with anticancer activities. Molecules.

[24] Umesalma S, Sudhandiran G (2010). Differential inhibitory effects of the polyphenol ellagic acid on inflammatory mediators NF-kappaB, iNOS, COX-2, TNF-alpha, and IL-6 in 1,2-dimethylhydrazine-induced rat colon carcinogenesis. Basic Clin Pharmacol Toxicol.

[25] You JS, Jones PA (2012). Cancer genetics and epigenetics: two sides of the same coin?. Cancer Cell.

[26] Zhang EH, Wang RF, Guo SZ, Liu B (2013). An update on antitumor activity of naturally occurring chalcones. Evid Based Complement Alternat Med.

